# Trend analysis and spatiotemporal distribution of leishmaniasis disease incidence in Sri Lanka: A detailed review from 2009 to 2023

**DOI:** 10.1371/journal.pntd.0013158

**Published:** 2025-07-02

**Authors:** Nayana Gunathilaka, Deshaka Jayakody, Rajitha Wickremasinghe

**Affiliations:** 1 Department of Parasitology, Faculty of Medicine, University of Kelaniya, Ragama, Sri Lanka; 2 Department of Public Health, Faculty of Medicine, University of Kelaniya, Ragama, Sri Lanka; Tulane University School of Public Health and Tropical Medicine, UNITED STATES OF AMERICA

## Abstract

**Background:**

Leishmaniasis, a significant emerging and re-emerging zoonotic disease, has exhibited a marked increase in incidence globally and in Sri Lanka. Analyzing its incidence in different geographical locations provides insights into its transmission dynamics for designing and implementing control interventions. This study aimed to investigate the disease trends in Sri Lanka at the national level using statistical models.

**Method:**

Weekly records of leishmaniasis cases from 2009 to 2023 were accessed from the weekly epidemiology reports published by the Epidemiology Unit of the Ministry of Health, Sri Lanka. The incidence rate (IR) for each year for 25 administrative districts in Sri Lanka was calculated per 100,000 population using estimated population data published by the Department of Census and Statistics, Sri Lanka. The IR was modeled using a Generalized Additive Model for Location, Scale, and Shape (GAMLSS). Data were analyzed using R 4.4.0 software and gamlss, mgcv, sp, ggplot2, dplyr, and rgl packages.

**Results:**

A total of 30,295 leishmaniasis cases were recorded, with Hambantota (68.32), Polonnaruwa (52.21), and Anuradhapura (40.30) districts reporting the highest IRs. These three districts, along with Matara (26.38), Matale (25.48), and Kurunegala (16.21), accounted for over 83% (*n* = 25,151) of reported cases. The IRs of Anuradhapura, Matale, Ratnapura, Kegalle, and Puttalam districts steadily increased over time after 2015 to almost 2.0 cases per 100,000 population by 2022. A seasonal variation is seen mid-year peaking around June to August.

**Conclusions:**

This study reveals temporally regressive and spatially expanding incidence of cutaneous leishmaniasis in Sri Lanka with characteristic geographical patterns and disease hotspots, highlighting the need for comprehensive control strategies to prevent further spread and mitigate the impact of leishmaniasis in the country.

## Background

Leishmaniasis, a neglected tropical disease caused by protozoan parasites of the genus *Leishmania,* is transmitted by the bite of female Phlebotomine sand flies (Diptera: Psychodidae) presenting as visceral, mucocutaneous, and cutaneous leishmaniasis (CL) [1]. About 21 species under the *Leishmania* genus have been identified as causative agents [2]. The parasite determines the clinical type of the infection. In general, *L. major*, *L. tropica*, *L. aethiopica*, and *L. infantum* cause old-world CL found in southern Europe, the Middle East, Asia, and Africa, respectively [3]. The causative agents causing Mucocutaneous Leishmaniasis (MCL) are *L. braziliensis*, *L. amazons*, *L. panamensis*, and *L. Guyanese* [3]. *Leishmania donovani* complex is mainly responsible for the visceral form of the disease [4]. However, species of *L. donovani* complex also cause other types of the disease [2,5].

Epidemiological reports available from the World Health Organization (WHO) revealed that over 85% of newly discovered CL are reported from Afghanistan, Algeria, Bolivia, Brazil, Colombia, Iran, Iraq, Pakistan, Syria, and Tunisia. In comparison, over 95% of newly discovered visceral leishmaniasis (VL) cases originated in Brazil, China, Ethiopia, India, Iraq, Kenya, Nepal, Somalia, and Sudan. Brazil, Bolivia, Ethiopia, and Peru have reported almost 90% of all new cases of MCL [3,6].

The first case of leishmaniasis in Sri Lanka was reported in 1904 and then cases were regularly reported till 1962 [7]. After about 30 years, the disease reappeared in 1990, first as an imported case and then as an indigenous case in 1992 in the Southern Province of Sri Lanka [8]. The non-reporting of leishmaniasis cases for over 30 years is postulated to be due to the widespread use of indoor residual spraying for malaria control. Since then, the incidence has spread to other parts of the country; hence, the Ministry of Health, Sri Lanka, declared leishmaniasis as a notifiable disease in 2008. An action plan was developed to control leishmaniasis in 2009 [9].

Currently, Sri Lanka is considered one of the endemic countries for CL [10] in the world and the newest reported focus of leishmaniasis in the Indian subcontinent, where the disease is caused by the most virulent visceralizing species, *L. donovani*. All three clinical forms of leishmaniasis have been identified in the country, and almost 99% of the case burden comes from CL cases [9].

Recently, leishmaniasis started to spread into other districts in the wet zone of the country, which were not previously considered endemic districts for disease transmission [11–13]. There were no leishmaniasis control activities formalized in the country or was a focal point within the Ministry of Health assigned this task; after the disease was made a notifiable disease, the Epidemiology Unit of the Ministry of Health issued guidelines on its control; since August 2022, the Anti Malaria Campaign (AMC), after successfully eliminating malaria, was appointed as the focal point for leishmaniasis control in Sri Lanka.

Given this scenario, a National Strategic Plan (NSP) for preventing and controlling leishmaniasis in Sri Lanka 2024–2028 was developed with WHO assistance. The goal of the NSP is to control CL for possible elimination in the future with the objectives of reducing the annual incidence of CL < 5 per 10,000 population by 2028 (a total of approximately 6,600 cases) and ensuring zero mortality due to VL. This plan comprises three strategic interventions (disease surveillance, diagnosis and case management, and integrated vector management) and five supportive areas (leadership, programme governance and management, community awareness and engagement on prevention/care, quality assurance, training, and operational research). Trend analysis of case incidence provides valuable insights into the disease transmission and spread over time. It also provides information for monitoring and evaluating past and current interventions, recognizing emerging hotspots, and predicting future outbreaks. Public health authorities can identify patterns and correlations with environmental, socio-economic, and climatic factors by analyzing trends, enabling a more targeted and timely response to CL outbreaks.

Understanding both the epidemiological trends and the underlying entomological and human risk factors is crucial for designing effective control strategies. The first record of the sand fly, *Phlebotomus argentipes,* in Sri Lanka dates back to 1910 [14]. The most recent update on the taxonomy of local sand fly fauna indicated the presence of 22 sand fly species, including the 3 sibling species in the *P. argentipes* complex [15,16]. *Phlebotomus argentipes*, the primary vector in Sri Lanka, has shown that peak biting activity typically occurs between 8:00 PM and 11:00 PM, indicating a predominantly nocturnal behavior [17]. While *P. argentipes* in Sri Lanka remains broadly susceptible to commonly used insecticides, early signs of resistance to malathion have been reported [18]. Regarding patient risk factors, children under 18 years of age and individuals engaged in outdoor occupations are at a notably higher risk of infection. Interestingly, people living near CL patients appear to face a higher infection risk than household members, likely due to differences in disease awareness and protective behavior [19]. These entomological and epidemiological insights are critical for contextualizing the observed spatial and temporal patterns of CL transmission, emphasizing the importance of integrating vector biology, insecticide management, and human behavioral factors into control strategies.

Spatiotemporal distribution patterns comprehensively understand how the disease spreads across different regions and periods. This analysis helps identify high-risk areas, understand the dynamics of disease transmission, and assess the impact of vector control measures. In a country like Sri Lanka, where geographical and climatic variations significantly influence disease transmission, analyzing the spatial distribution of leishmaniasis can highlight areas needing intensified surveillance and intervention; it will provide critical insights necessary for prioritizing areas that need to be targeted, optimizing disease control efforts, improving public health responses, and ultimately reducing the burden of leishmaniasis in the country.

Several epidemiological studies have assessed the prevalence, distribution, and transmission dynamics of leishmaniasis in Sri Lanka, yet comprehensive spatial and temporal analyses remain limited [20]. It has been highlighted that the geographic expansion of leishmaniasis [11], particularly in previously non-endemic regions [21], and the emergence of visceral leishmaniasis, raising concerns about its underreporting and potential misdiagnosis [22]. However, systematic epidemiological trend analyses have not been extensively conducted. Therefore, this study aimed to use advanced statistical models to analyze the national data available in the public domain to determine the spatial and temporal distribution of cases and predict leishmaniasis incidence. This study fills this critical gap by employing advanced statistical models to analyze available public domain data, mapping the spatial and temporal distribution of cases, and predicting future incidence trends. Such an approach is essential for identifying high-risk areas, optimizing resource allocation, and enhancing targeted intervention strategies, ultimately strengthening national leishmaniasis control efforts.

## Method

### Study area

Sri Lanka is a tropical island in the Indian Ocean off the southern tip of the Indian peninsula. The island has an area of approximately 65,610 km^2^ and has diverse habitats with a complex topography and variable rainfall patterns [23]. Administratively, Sri Lanka is divided into 25 districts. Geographically, it has mostly flat to rolling coastal plains with mountainous regions in the south-central part of the island, where the highest elevation reaches 2,524 m above mean sea level. Rainfall patterns are influenced by seasonal monsoonal winds, with annual precipitation measuring about 2,500 mm in the central highlands, and around 1,200 mm in the dry zones of the east, southeast, and northern parts of the country. The semi-arid northwest and southeast coasts receive between 800 and 1,200 mm of rainfall annually. The mean temperature ranges from 17 °C in the highlands to 33 °C in the coastal areas. According to the latest United Nations data, the estimated population is projected to be 21,944,624 [24].

### Collection of data

Weekly records of leishmaniasis cases recorded from January 2009 to December 2023 in each administrative district were accessed from the weekly epidemiology reports archived in the official website of the Epidemiology Unit of the Ministry of Health, Sri Lanka. The mid-year population data for each year for each district during the corresponding time was obtained from the Department of Census and Statistics of Sri Lanka. The meteorological parameters, namely mean temperature, maximum temperature, minimum temperature, total rainfall, and mean relative humidity for each month by district and year were obtained using the nasapower package in R version 4.4.0. The nasapower package retrieves data from the NASA Prediction of Worldwide Energy Resources database, which provides satellite-derived meteorological variables [25].

### Data analysis

#### Incidence rate calculations.

The incidence rates, confidence intervals, and district- and year-specific average incidence rates were calculated using the formulas given in supplementary information (S1 Table).

#### Modeling of leishmaniasis incidence.

Leishmaniasis incidence rates were analyzed using a Zero-Adjusted Gamma (ZAGA) family within the Generalized Additive Model for Location, Scale, and Shape (GAMLSS). In this model, the incidence rate was treated as the response variable. This framework was selected specifically to address the prominent feature of zero inflation in the leishmaniasis incidence rate. The ZAGA family was selected to account for the excess zeros observed in the incidence rate data, as 58.56% of the cases reported zero incidence rates. Environmental parameters, including mean relative humidity (RH), mean temperature, maximum temperature, minimum temperature, and total rainfall, were included as predictor variables. Latitude and longitude of the center of districts were used to model spatial effects, while year and month were included as temporal variables to account for temporal variations in the incidence rates. The ZAGA spatial GAMLSS model is defined by three linked equations, which model the expected incidence rate (*μ*), dispersion (*α*), and zero-inflation probability (*v*) as smooth functions of the predictors. These components are described by the following equations:

(1) Expected incidence rate model

$$\begin{array}{cc} \log\left(\mu_{i}\right) & =\beta_{0}+f_{1}({mean\;relative\;humidity}_{i})+f_{2}(mean\;t{emperature}_{i})+f_{3}({maximum\;temperature}_{i})\\ & +f_{4}(m{inimum\;temperature}_{i})+f_{5}({total\;rainfall}_{i})+f_{6}(y{ear}_{i})+f_{7}(m{onth}_{i})+f_{8}(l{atitude}_{i})+f_{9}({logitude}_{i}) \end{array}$$

(2) Dispersion model

$$\begin{array}{cc} \log\left(\alpha_{i}\right) & =\;\gamma_{0}+\;f_{1}({mean\;relative\;humidity}_{i})+\;f_{2}(mean\;t{emperature}_{i})+\;f_{3}({maximum\;temperature}_{i})\\ & +\;f_{4}({mimimum\;temperature}_{i})+\;f_{5}({total\;rainfall}_{i}) \end{array}$$

(3) Zero-inflation model

$$\log\left(\frac{v_{i}}{1-v_{i}}\right)=\;\theta_{0}+\;g_{1}({latitude}_{i})+\;g_{2}({longitude}_{i})$$

For district “*i*,”

$\log\left(\mu_{i}\right)$ = log-transformed expected incidence rate

$\log\left(\alpha_{i}\right)$ = log-transformed dispersion parameter of the Gamma distribution

$\log\left(\frac{v_{i}}{1-v_{i}}\right)$= log-odds of zero incidence due to zero-inflation.

$\beta_{0},\gamma_{0}$ ${and\;\theta }_{0}$ are intercept terms. $f_{1}$ through $f_{9}$ and $g_{1},\;g_{2}$ represent smooth functions.

The goodness of fit of the model was assessed by examining the distribution of the residuals. All data were analyzed using R 4.4.0 software, employing the gamlss, mgcv, sp, ggplot2, dplyr, and rgl packages.

## Results

### Cutaneous leishmaniasis case incidence

**Fig 1:**
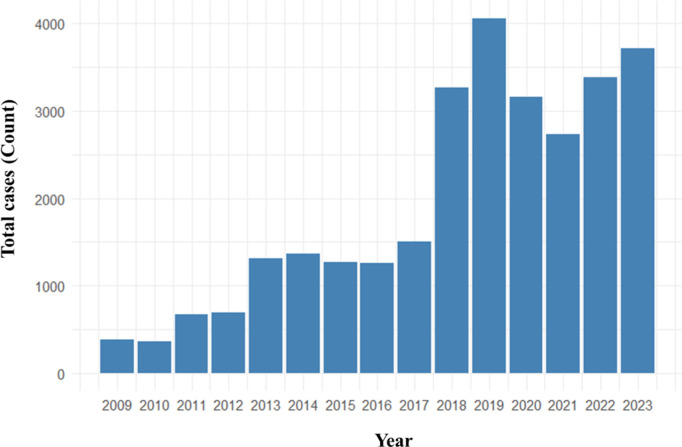
Distribution of leishmaniasis cases reported from 2009 to 2023 by year.

**Fig 2:**
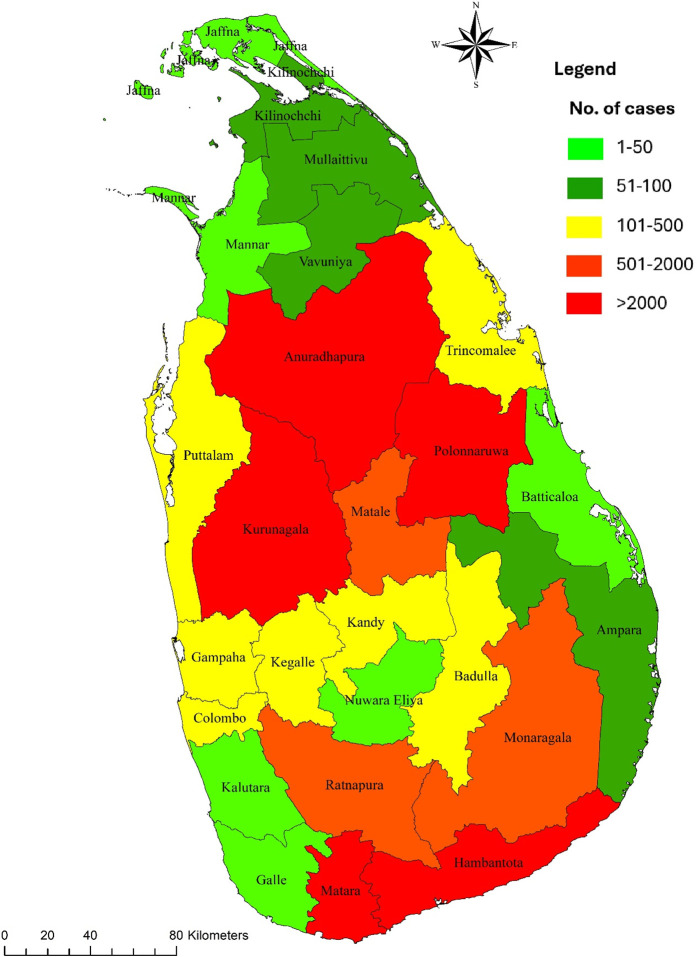
Reported cases of leishmaniasis from 2009 to 2023. (https://data.humdata.org/dataset/sri-lanka-administrative-levels-0-4-boundaries).

The CL cases reported during 2009–2023 are illustrated in Fig 1. During this period, 30,295 leishmaniasis cases were reported in Sri Lanka. There has been a more than 2-fold increase in the incidence since 2017. From 2022 onwards, the number of notifications has risen (Fig 1). During the period 2009–2023, the average annual incidence rate per 100,000 population was highest in Hambantota district (68.32), followed by Polonnaruwa (52.21), Anuradhapura (40.30), Matara (26.38), Matale (25.48), and Kurunegala (16.21) districts. The annual incidence rates (per 100,000 population per year) of leishmaniasis in different districts of Sri Lanka from 2009–2023 (S2 Table) and the total number of reported cases by district from 2009 to 2023 S1 Fig) are provided as supporting information, and the spatial map of case distribution is presented in Fig 2.

### Key predictor effects in ZAGA GAMLSS mode

The expected incidence rate (μ) was significantly influenced by several factors (Table 1). Mean temperature had a significant positive effect (estimate = 0.27: *P* < 0.05) suggesting that higher mean temperatures are associated with increased incidence rates. The maximum temperature (estimate = −0.12, *P* < 0.05) and minimum temperature (estimate = −0.14: *P* < 0.05) had a negative effect. The dispersion model estimated the variability in incidence rates across districts (Table 2). It showed that total rainfall was significantly positively related to lower variability in incidence rates (estimate = 0.00: *P *= 0.04). Other environmental variables such as mean temperature, maximum temperature, minimum temperature and mean RH were not significant in the dispersion of incidence rates. The zero-inflation model examined the probability of excess zeros in the incidence data (Table 3). Latitude was positively associated with excess zeros in incidence rates (estimate = 0.50: *P* = 0.00), whereas longitude had a negative effect (estimate = −0.35: *P* = 0.00).

**Table 1:** Parameter and risk estimates of the expected incidence rate model.

Variable	Estimate	Std. error	Risk estimate	95% Confidence interval of risk estimate	t-value	*P*-value
(Intercept)	−312.10	0.34	–	–	−927.60	0.00
pb (mean relative humidity)	0.00	0.00	1.00	0.99–1.01	0.81	0.42
pb (mean temperature)	0.27	0.06	1.31	1.18–1.43	4.13	0.00
pb (maximum temperature)	−0.12	0.03	0.89	0.83–0.94	−4.15	0.00
pb (minimum temperature)	−0.14	0.04	0.87	0.79–0.94	−3.67	0.00
pb (total rainfall)	0.00	0.00	1.00	0.99–1.00	−0.85	0.39
pb (year)	0.12	0.00	1.12	1.12–1.12	601.01	0.00
pb (month)	0.01	0.01	1.02	1.00–1.03	2.90	0.00
pb (latitude)	0.18	0.02	1.19	1.16–1.23	10.21	0.00
pb (longitude)	0.97	0.01	2.63	2.61–2.64	133.27	0.00

**Table 2:** Parameter and risk estimates of the dispersion model.

Variable	Estimate	Std. error	Dispersion ratio	95% Confidence interval of risk estimate	*t*-value	*P*-value
(Intercept)	−0.56	0.90	–	–	−0.62	0.53
pb (mean relative humidity)	0.00	0.01	1.00	0.99–1.01	0.65	0.51
pb (mean temperature)	−0.02	0.04	0.98	0.90–1.05	−0.58	0.56
pb (maximum temperature)	0.02	0.02	1.02	0.97–1.05	0.80	0.43
pb (minimum temperature)	0.00	0.02	1.00	0.95–1.04	−0.12	0.91
pb (total rainfall)	0.00	0.00	1.00	1.00–1.00	2.04	0.04

**Table 3:** Parameter and risk estimates of the zero-inflation model.

Variable	Estimate	Std. error	Odds ratios	95% Confidence interval of risk estimate	*t*-value	*P*-value
(Intercept)	24.52	4.03	–	–	6.09	0.00
pb (latitude)	0.50	0.04	1.65	1.57–1.73	12.04	0.00
pb (longitude)	−0.35	0.05	0.71	0.61–0.81	-6.94	0.00

### Goodness of fit of the model

**Fig 3:**
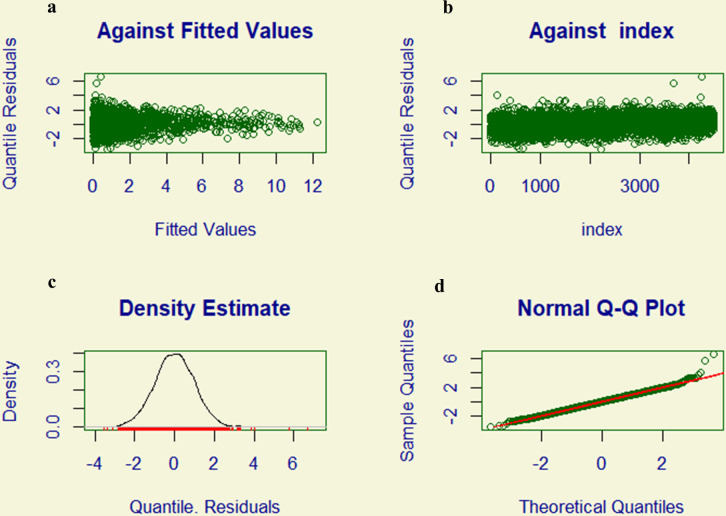
Residual diagnostic plots for assessing the adequacy of the model. Zero-adjusted gamma (ZAGA) generalized additive model for location, scale, and shape (GAMLSS). **(a)** Residuals plotted against fitted values; **(b)** Residuals plotted against the index; **(c)** Density estimates of the residuals; and **(d)** Normal Q–Q plot.

The residual diagnostic plots for the ZAGA GAMLSS model suggest a good fit to the data. Fig 3a shows residuals randomly scattered around zero against fitted values, indicating no major issues with non-linearity or heteroscedasticity. Similarly, Fig 3b reveals no discernible patterns in residuals plotted against the index, suggesting a lack of significant autocorrelation. The density estimates in Fig 3c indicates that the residuals are approximately normally distributed as a bell-shaped curve. The Normal Q–Q plot in Fig 3d further supports the normality assumptions as the residuals closely follow the diagonal line. Collectively, these plots do not reveal critical violations of model assumptions, suggesting that the ZAGA GAMLSS model adequately captures the data’s characteristics.

### Temporal distribution of leishmaniasis disease incidence

**Fig 4:**
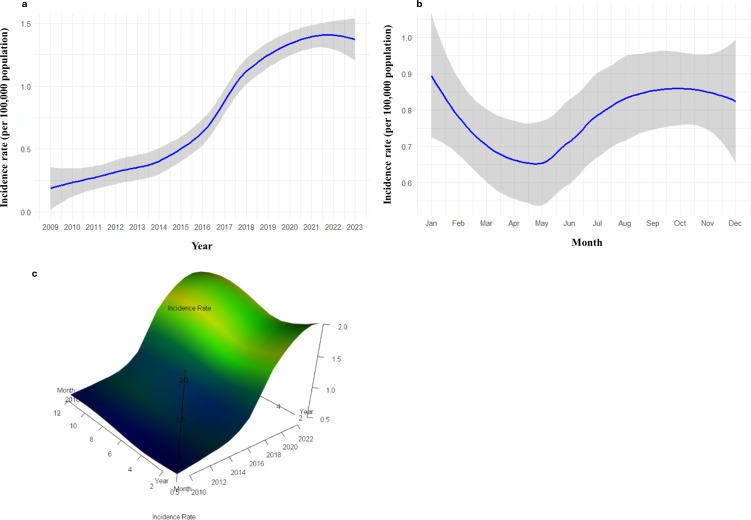
Fitted incidence rates per 100,000 population in Sri Lanka from 2009 to 2023, modeled using zero-adjusted gamma (ZAGA) generalized additive model for location, scale, and shape (GAMLSS). **(a)** Yearly trend of incidence rate; **(b)** seasonal trend of incidence rate; and **(c)** 3D surface visualization of fitted incidence rate by month and year.

The year (estimate = 0.12: *P* = 0.00) and month (estimate = 0.01; *P* = 0.00) showed significant positive effects indicating an increasing trend in the incidence rate over time and seasonality. Fig 4a shows the trend of CL incidence rates in Sri Lanka from 2009 to 2023. It illustrates a steady increase in incidence rates over time, particularly after 2015, reaching close to 2.0 per 100,000 persons per year by 2022. The shaded area represents the confidence interval, indicating the reliability of the fitted line. The incidence rate is lowest around April and gradually increases, peaking around September before slightly declining towards December (Fig 4b). The shaded area around the line represents the confidence interval, indicating the range of uncertainty in the fitted rates.

A 3D representation of the monthly and annual incidence of leishmaniasis cases in Sri Lanka indicates a clear upward trend in the incidence rate over the years, indicating that the overall burden of CL has been increasing over the period of study (Fig 4c). Monthly variation is also evident, with certain months having higher incidence rates than others, suggesting potential seasonality in the occurrence of CL cases. The year–month interaction had varying incidence rates, with more recent years experiencing higher rates across most months compared to earlier years, indicating that the increase in CL incidence is not limited to specific months but is a generally an upward trend over time.

### Spatial distribution of leishmaniasis disease incidence

**Fig 5:**
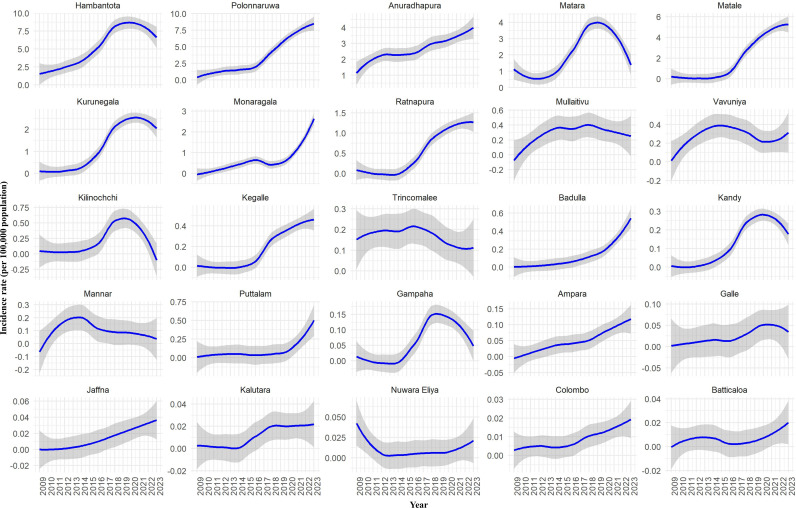
Fitted incidence rates of leishmaniasis per 100,000 population by year and districts using a generalized additive model for location, scale, and shape (GAMLSS) model with a zero-adjusted gamma (ZAGA) distribution (2009–2023).

**Fig 6:**
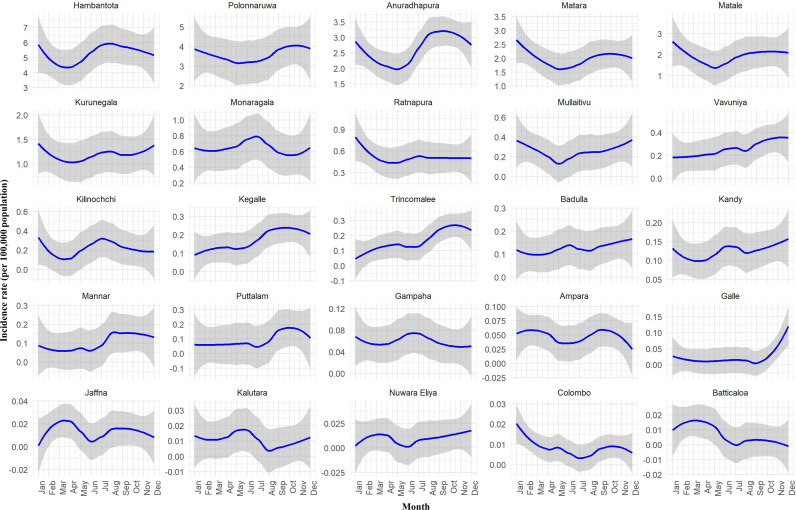
Fitted incidence rates of leishmaniasis per 100,000 population by month and districts using a generalized additive model for location, scale, and shape (GAMLSS) model with a zero-adjusted gamma distribution (ZAGA) (2009–2023).

The fitted incidence rates of CL per 100,000 persons across various districts in Sri Lanka from 2009 to 2023 is illustrated in Fig 5. Each subplot represents a different district, displaying trends in the incidence rates over the years. Anuradhapura, Matale, Ratnapura, Kegalle, Badulla, Ampara, Colombo, and Puttalam districts showed a steady increase in incidence rates (Fig 5). The incidence rate of CL in Hambantota, Gampaha, and Kurunegala districts peaked around 2020 with a subsequent decline. Monaragala and Polonnaruwa districts had a significant upward trend starting around 2017/2018. In Matara and Kilinochchi districts, the incidence rate peaked around 2018 with a subsequent decline later on. The CL incidence rates in Mullaitivu, Trincomalee, Vavuniya, Nuwara Eliya, and Batticaloa districts remain low and stable with minor fluctuations. In Galle and Kalutara districts, the incidence rate peaked around 2018/19 and then stabilized. There were minor fluctuations in the other districts. The seasonal variation of each district is illustrated in Fig 6.

## Discussion

Leishmaniasis, especially CL, is emerging in new geographic areas and re-emerging in others; however, visceral leishmaniasis is targeted for elimination as a public health problem by the end of 2026 [26]. In Sri Lanka, there was an absence of reports on leishmaniasis for about 30 years from 1962 onwards [7], and this disease was considered an emerging disease that was reported in the 1990s [8]. In 2008, leishmaniasis was declared a notifiable disease in Sri Lanka; no formalized control programme has been initiated as yet except for the diagnosis and treatment of cases reported to hospitals.

In this study, we focused on identifying the spatial and temporal trends of leishmaniasis incidence from 2009 to 2023 based on archived records of the Epidemiology Unit of the Ministry of Health, Sri Lanka, after it was declared a notifiable disease. The incidence of leishmaniasis has increased in many countries [27]. In 2018, over 253,000 new CL cases were reported to the World Health Organization [28], a similar trend has been observed in Sri Lanka, with a more than 2-fold increase after 2017. Early studies have indicated that the incidence was low and sporadic before 2001 [29], a 15-fold increase in cases was observed from 2001 to 2010 for many reasons [30]. This study revealed that the incidence rates, especially in the recent years after 2015, have increased drastically, highlighting the need for intensified monitoring and interventions. The disease trends highlight specific periods where the incidence rate peaks, especially in recent years, which can help in identifying critical periods for intensified monitoring and intervention. Overall, the 3D visualization in this study effectively captures the increasing trend of CL incidence in Sri Lanka, with nuances in seasonal variation, emphasizing the need for ongoing surveillance and targeted public health strategies throughout the year.

Since becoming a notifiable disease, the vigilance for the disease may likely have improved diagnosis and treatment facilities, increased awareness among the community and clinicians would positively influence health-seeking behaviors of the community and case notification. In Sri Lanka, the malaria control programme used indoor residual insecticide spraying (IRS) targeting adult vectors. Changing strategies in the mid-1990s to focal spraying was initiated with a further gradual decrease in malaria cases since 2000. Gradual withdrawal of IRS for malaria control may have played a role in the emergence of leishmaniasis since the 1990s [21]. Leishmaniasis control is widely recognized as a beneficial by-product of the region’s concerted malaria control efforts [31]. This shift presents an almost mirror-image scenario, highlighting the intricate dynamics between these two diseases having a standard control measure. Therefore, the recent increasing trend of leishmaniasis could be due to the rebounding effect of withdrawing IRS activities after eliminating malaria. Although AMC has been assigned as the national focal point for the control of leishmaniasis in Sri Lanka, no vector data are available as yet, making it impossible to evaluate the impact of the cessation of IRS on vector biology. As a result, a comprehensive understanding of vector population dynamics remains limited. Another major drawback is the lack of awareness about the disease among the general public, as well as healthcare professionals, coupled with treatment failures. Therefore, awareness programmes should be implemented to address these challenges.

The spatiotemporal model is a powerful tool for detecting transmission hotspots and understanding the driving forces behind disease transmission. For example, how spatial dispersal and locally correlated climatic variables contribute to transmission [32–37]. While earlier studies [11,21] provided important baseline knowledge, they were limited by shorter time spans, smaller geographic focus, and primarily descriptive or hotspot mapping methods. In contrast, this study analyzes national surveillance data over a 15-year period from 2009 to 2023, extending well beyond the scope of prior publications to capture recent epidemiological shifts, including post-2018 case surges in emerging districts such as Matale and Kegalle. Moreover, an advanced statistical modeling framework GAMLSS with a ZAGA distribution was employed to model spatial and temporal patterns in a more robust and predictive manner, explicitly accounting for zero-inflated incidence data. This approach, not previously used in Sri Lankan leishmaniasis research, enables deeper insights into the relationship between incidence trends and climatic factors and allows to identify evolving disease hotspots with greater precision. These enhancements provide timely, policy-relevant evidence that directly aligns with and supports the objectives of the National Strategic Plan (2024–2028).

In this study, the spatiotemporal model predicted that cases will continue to rise unless viable interventions are implemented. A similar trend has been iterated in previously published studies [30,38]. However, the low patient notifications in 2020/2021 may be due to travel restrictions and lockdowns imposed during the COVID-19 pandemic that influenced the treatment-seeking behavior of infected individuals and restrictions on activities that could minimize the potential exposure to risk factors of the disease [13].

Overall, 83.02% (*n* = 25,151) of the cases reported from the country were from six districts (Hambantota, Polonnaruwa, Anuradhapura, Matara, Matale, and Kurunegala). Therefore, immediate attention should be drawn to control disease transmission in these districts. Even though leishmaniasis was more or less confined to only districts in the dry zone of the country earlier, recent evidence of the spread of the disease reveals new foci bordering endemic districts in the wet zone of the country (Gampaha and Kegalle) is emerging [11–13]. “A similar trend has been observed from the newly emerging disease foci in Sri Lanka, such as Matale, Ratnapura, and Moneragala districts bordering high disease endemic districts. Establishing new foci in proximity to high disease endemic districts may be facilitated through population movements from high endemic districts, the presence of similar environmental conditions and other risk factors favorable to disease transmission and vector abundance in these areas [13]. Overall, the present study indicates that locations at more northern latitudes are more likely to experience lower incidence rates, whereas districts with more eastern longitudes are likely to have a higher case incidence.”

Additionally, population movements from endemic to non-endemic districts may have facilitated the establishment of the disease in new areas, especially in border areas [13]. Therefore, analysis of disease progression in an area at its early stage is essential in preventing disease establishment and implementing appropriate interventions among risk communities. This will be beneficial to minimize the further spread of the disease into other areas [13]. Overall, the data indicated varied trends across different districts, with some showing significant increases in incidence rates. In contrast, others remain stable or in decline, suggesting that CL incidence is highly localized and requires targeted interventions based on specific district trends.

Infectious disease outbreaks interact with spatial and temporal dimensions [39]. It is known that spatial heterogeneity is subject to differences in the distribution of vectors and other risk factors. Further, it influences shifting the patterns of vector–parasite interactions, vector–host contact, and the population’s susceptibility [40]. In Sri Lanka, climatic conditions have been shown to affect the occurrence of vector-borne diseases such as malaria and dengue [41,42]. A time series analysis of leishmaniasis incidence in Sri Lanka and climatic factors have indicated that RH is the best predictive climatic variable [38]. However, RH is determined by several associated environmental/climatic variables such as temperature and rainfall. This study indicated that higher rainfall may lead to more stable patterns of incidence rates across districts. Further, both maximum and minimum temperatures denoted negative effects indicating that extremes of heat reduce incidence rates.

In this study, it is seen that the seasonality of leishmaniasis incidence in Sri Lanka peaks in September and October with a trough in April. This could be attributed to the climatic factors and their impact on vector ecology. Sri Lanka experiences two main monsoon seasons: the southwest monsoon from May to September and the northeast monsoon from December to February. The months of September and October fall at the end of the southwest monsoon, characterized by increased convectional rainfall and humidity, which create favorable breeding conditions for *P. argentipes*, the primary sand fly vector in the country. These climatic conditions enhance the survival and reproduction of sand flies, leading to a rise in their population and the transmission of *Leishmania* parasites.

Conversely, April marks the end of the dry season, typically resulting in reduced sand fly activity and lower transmission rates. During this period, the dry and hot conditions are less conducive to sand fly breeding and survival, leading to decreased leishmaniasis cases. Thus, the seasonality of leishmaniasis incidence in Sri Lanka is closely linked to the climatic patterns and their influence on vector dynamics.

A major limitation of this study is the reliance on weekly records of leishmaniasis cases reported by the Epidemiology Unit, Ministry of Health, Sri Lanka; data accuracy depends on accuracy and coverage of notification and reporting. We believe the number of cases reported is a gross under-estimate of the actual numbers, but this inaccuracy will unlikely affect the increasing trends described in this manuscript. Additionally, we did not investigate potential confounding factors such as changes in health-seeking behavior, public awareness, and diagnostic practices, which could have influenced the number of cases reported and the observed trends. The absence of potential confounding factors, such as changes in health-seeking behaviors, public awareness, and diagnostic practices, may have influenced model outcomes. Increased awareness and improved healthcare access over time could lead to higher case detection, while limited access in certain districts may result in under-reporting, introducing bias in incidence estimates. These unaccounted factors may also contribute to spatial and temporal inconsistencies, impacting the accuracy of risk estimates. Future studies incorporating healthcare accessibility and reporting trends could provide a more comprehensive understanding of the true environmental drivers of leishmaniasis. The significant zero-inflation in the incidence data suggests that the model should have included unmeasured factors, possibly related to disease prevalence or inconsistencies in reporting practices across districts. Lastly, the absence of a well-planned designated control programme for leishmaniasis in Sri Lanka poses challenges in consistently and effectively implementing control measures across the country. For this study, detailed and reliable data on such populations were not consistently available across all districts and years within our study period. Therefore, to ensure consistency and feasibility, we used the estimated mid-year population as the denominator for calculating incidence rates. While this approach is less precise than using a specific at-risk population, it provides a standardized and readily available denominator for comparing incidence rates across districts and over time. Furthermore, inter-district mobility, particularly seasonal labor migration, could introduce a degree of bias into our incidence rate calculations. Individuals may be estimated in the mid-year population of one district but may have acquired leishmaniasis in another. While we recognize this limitation, detailed data on inter-district mobility patterns and their specific relevance to leishmaniasis transmission were not available for our study area and period. Therefore, using the mid-year population, while imperfect, provided a consistent and broad representative denominator across all districts and years, aligning with common practices in ecological epidemiological studies.

A key limitation of this study is the absence of a specific temporal autocorrelation structure, such as Distributed Lag Nonlinear Models (DLNMs), within our GAMLSS framework. While DLNMs could provide a more detailed representation of lagged effects and nonlinear relationships between environmental drivers and disease incidence, their integration would have significantly increased model complexity and computational demands. Additionally, the primary objective of this study was to assess broader spatiotemporal patterns and distributional characteristics of incidence rates rather than to dissect intricate lagged dependencies. Our approach, which employs smooth functions for temporal variables within GAMLSS, offers a robust and interpretable initial assessment of trends and seasonality. However, future studies designed explicitly to explore lagged environmental effects using DLNMs could further enhance our understanding of temporal dependencies in disease transmission dynamics.

In Sri Lanka, leishmaniasis is typically diagnosed through microscopic examination of tissue samples, such as from skin lesions, and PCR-based techniques for more accurate detection of the Leishmania parasites. However, reporting bias is a concern, as not all individuals with leishmaniasis seek formal medical treatment. Many may opt for home remedies or alternative therapies instead of visiting healthcare facilities, leading to underreporting of cases. This can result in an inaccurate representation of the disease burden, which may skew epidemiological data and hinder targeting of effective control measures.

This study provides a foundation for informing leishmaniasis control in Sri Lanka. The model’s insights into the spatial and environmental drivers of disease incidence highlight key areas where public health efforts, such as targeted vector control and surveillance, can be intensified. Additionally, the study underscores the need for further research into vector dynamics and environmental factors, providing a basis for more effective interventions and evidence-based decision-making in the fight against leishmaniasis.

Future studies should focus on a more detailed characterization of vector species, such as *P. argentipes*, including their distribution, behavior, and insecticide resistance patterns. Additionally, genetic and molecular analyses of *L. donovani* complexes could provide deeper insights into parasite diversity, transmission dynamics, and potential variations in virulence. Such research could enhance surveillance strategies and inform targeted vector control and treatment interventions, ultimately improving leishmaniasis management in Sri Lanka.

## Conclusions

There is a significant increase in leishmaniasis incidence in Sri Lanka from 2009 to 2023. Hambanthota, Polonnaruwa, Anuradhapura, Matara, Matale, and Kurunegala districts reported the highest incidence rates, collectively accounting for over 83% of cases countrywide. There were significant year and seasonal trends. The incidence peaks in September and October, aligning with the end of the southwest monsoon, emphasizing the role of environmental conditions in vector dynamics. Based on the spatiotemporal model used in this study, the incidence and the spread of CL is likely to increase in the country; immediate, targeted public health interventions and an enhanced disease surveillance system are imperative to control the spread and mitigate the impact of leishmaniasis in Sri Lanka in the next few years.

## Supporting information

S1 Table Detailed formulas used to calculate incidence rates, confidence intervals, and average incidence measures in the study. (DOCX)

S2 Table Annual incidence rates (per 100,000 population per year) of leishmaniasis in different districts of Sri Lanka, 2009–2023. (DOCX)

S1 Fig Distribution of total leishmaniasis cases reported from each adminstrative district in Sri Lanka from 2009 to 2023. (TIFF)
