# Supplementary material for: Trend analysis and spatiotemporal distribution of leishmaniasis disease incidence in Sri Lanka: A detailed review from 2009 to 2023
Source: PLoS Negl Trop Dis. 2025 Jul 2;19(7):e0013158. doi: 10.1371/journal.pntd.0013158 (PMC12221050; doi:10.1371/journal.pntd.0013158)
Supplement: S1 Table — (DOCX) [file pntd.0013158.s001.docx]

| **Calculation** | **Formula** |
| --- | --- |
| Incidence rate | $\frac{Total Number of new cases}{Total number at risk} \times100 000$ $\frac{Total Number of new cases}{Total number at risk} \times100 000$  (the total number at risk was taken as the mid-year population of the district for each year during the period) |
| 95 % Confidence interval of the incidence rate | $p \pm1.96 \sqrt{\frac{p*(1-p)}{risk population}}$ |
| District Average Incidence Rate (2009-2023) | $\frac{\sum_{2009}^{2023} {Incidence rate}_{\mathrm{year}}}{15}$ |
| Yearly Average Incidence Rate (Across 25 districts) | $\frac{\sum_{1}^{25} {Incidence rate}_{\mathrm{District}}}{25}$ |

**Supplementary Table S1**: Detailed formulas used to calculate incidence rates, confidence intervals, and average incidence measures in the study
