# Supplementary material for: Trend analysis and spatiotemporal distribution of leishmaniasis disease incidence in Sri Lanka: A detailed review from 2009 to 2023
Source: PLoS Negl Trop Dis. 2025 Jul 2;19(7):e0013158. doi: 10.1371/journal.pntd.0013158 (PMC12221050; doi:10.1371/journal.pntd.0013158)
Supplement: S2 Table — (DOCX) [file pntd.0013158.s002.docx]

| **District** | **Year** | | | | | | | | | | | | | | | **District average** |
| --- | --- | --- | --- | --- | --- | --- | --- | --- | --- | --- | --- | --- | --- | --- | --- | --- |
|  | **2009** | **2010** | **2011** | **2012** | **2013** | **2014** | **2015** | **2016** | **2017** | **2018** | **2019** | **2020** | **2021** | **2022** | **2023** |  |
| Colombo | 0.00±0.00 | 0.09±0.12 | 0.04±0.08 | 0.13±0.15 | 0.04±0.08 | 0.13±0.14 | 0.04±0.08 | 0.00±0.00 | 0.04±0.08 | 0.21±0.18 | 0.25±0.20 | 0.12±0.14 | 0.04±0.08 | 0.20±0.18 | 0.28±0.21 | 0.11±0.11 |
| Gampaha | 0.04±0.09 | 0.09±0.12 | 0.00±0.00 | 0.04±0.09 | 0.22±0.19 | 0.13±0.15 | 0.13±0.14 | 0.29±0.22 | 0.33±0.23 | 2.82±0.67 | 7.20±1.07 | 2.48±0.63 | 0.53±0.29 | 1.89±0.55 | 1.98±0.56 | 1.21±0.33 |
| Kalutara | 0.00±0.00 | 0.00±0.00 | 0.08±0.16 | 0.08±0.16 | 0.00±0.00 | 0.00±0.00 | 0.00±0.00 | 0.00±0.00 | 0.08±0.15 | 0.70±0.46 | 0.23±0.26 | 0.00±0.00 | 0.00±0.00 | 0.31±0.30 | 0.39±0.34 | 0.13±0.12 |
| Kandy | 0.00±0.00 | 0.00±0.00 | 0.15±0.20 | 0.00±0.00 | 0.36±0.32 | 0.36±0.31 | 1.20±0.57 | 0.91±0.49 | 1.17±0.56 | 2.79±0.85 | 4.20±1.05 | 5.33±1.17 | 2.33±0.77 | 3.67±0.97 | 2.63±0.83 | 1.67±0.54 |
| Matale | 2.06±1.28 | 0.21±0.40 | 1.03±0.90 | 4.95±1.98 | 2.68±1.46 | 6.45±2.24 | 7.19±2.35 | 4.92±1.93 | 2.33±1.32 | 40.66±5.49 | 55.17±6.37 | 66.29±6.96 | 54.15±6.27 | 66.23±6.93 | 67.81±7.04 | 25.48±3.53 |
| Nuwara Eliya | 0.70±0.62 | 0.14±0.28 | 0.00±0.00 | 0.00±0.00 | 0.00±0.00 | 0.00±0.00 | 0.27±0.37 | 0.00±0.00 | 0.00±0.00 | 0.00±0.00 | 0.13±0.26 | 0.13±0.25 | 0.13±0.25 | 0.13±0.25 | 0.38±0.43 | 0.13±0.18 |
| Galle | 0.09±0.18 | 0.00±0.00 | 0.00±0.00 | 0.09±0.18 | 0.28±0.32 | 0.28±0.31 | 0.27±0.31 | 0.27±0.31 | 0.18±0.25 | 0.44±0.39 | 0.44±0.39 | 0.53±0.42 | 0.17±0.24 | 0.00±0.00 | 0.26±0.30 | 0.22±0.24 |
| Hambantota | 16.00±3.20 | 18.34±3.43 | 33.67±4.64 | 31.84±4.52 | 60.18±6.21 | 60.68±6.14 | 51.52±5.63 | 61.51±6.11 | 79.13±6.85 | 114.35±8.19 | 122.54±8.44 | 113.77±8.09 | 77.37±6.63 | 84.14±6.89 | 99.71±7.51 | 68.32±6.16 |
| Matara | 16.34±2.78 | 3.93±1.36 | 7.74±1.91 | 7.00±1.82 | 13.02±2.48 | 11.30±2.28 | 19.17±2.96 | 23.23±3.24 | 23.85±3.28 | 59.21±5.15 | 73.00±5.70 | 46.54±4.54 | 39.40±4.16 | 28.83±3.56 | 23.13±3.20 | 26.38±3.23 |
| Jaffna | 0.00±0.00 | 0.00±0.00 | 0.00±0.00 | 0.00±0.00 | 0.00±0.00 | 0.17±0.33 | 0.00±0.00 | 0.17±0.33 | 0.00±0.00 | 0.49±0.55 | 0.00±0.00 | 0.48±0.55 | 0.32±0.44 | 0.32±0.44 | 0.48±0.54 | 0.16±0.21 |
| Kilinochchi | 0.88±1.73 | 0.00±0.00 | 0.00±0.00 | 0.00±0.00 | 12.33±6.46 | 9.32±5.51 | 0.00±0.00 | 0.00±0.00 | 2.42±2.74 | 7.14±4.67 | 11.63±5.88 | 10.00±5.44 | 0.75±1.47 | 1.48±2.05 | 0.00±0.00 | 3.73±2.40 |
| Mannar | 0.00±0.00 | 0.00±0.00 | 0.00±0.00 | 2.01±2.78 | 4.02±3.94 | 4.90±4.30 | 0.97±1.90 | 0.00±0.00 | 0.00±0.00 | 3.67±3.60 | 0.90±1.77 | 0.89±1.75 | 0.88±1.72 | 0.00±0.00 | 0.86±1.69 | 1.27±1.56 |
| Vavuniya | 1.16±1.61 | 0.58±1.14 | 0.00±0.00 | 5.23±3.42 | 9.88±4.70 | 3.37±2.70 | 4.42±3.06 | 4.37±3.03 | 5.98±3.53 | 6.95±3.78 | 2.12±2.07 | 0.52±1.03 | 1.03±1.43 | 2.05±2.01 | 6.63±3.61 | 3.62±2.47 |
| Mullaiivu | 0.00±0.00 | 0.00±0.00 | 0.00±0.00 | 2.17±3.01 | 17.35±8.50 | 7.37±5.46 | 9.38±6.12 | 6.19±4.95 | 5.21±4.57 | 2.08±2.89 | 8.25±5.72 | 7.14±5.29 | 0.00±0.00 | 4.04±3.96 | 8.08±5.60 | 5.15±3.74 |
| Batticaloa | 0.00±0.00 | 0.00±0.00 | 0.00±0.00 | 0.38±0.53 | 0.00±0.00 | 0.00±0.00 | 0.00±0.00 | 0.18±0.36 | 0.18±0.35 | 0.00±0.00 | 0.00±0.00 | 0.17±0.34 | 0.00±0.00 | 0.34±0.47 | 0.17±0.34 | 0.09±0.16 |
| Ampara | 0.00±0.00 | 0.00±0.00 | 0.15±0.30 | 0.00±0.00 | 0.62±0.60 | 1.80±1.02 | 0.45±0.50 | 1.31±0.86 | 0.99±0.74 | 0.42±0.47 | 0.69±0.60 | 1.09±0.75 | 1.99±1.01 | 1.99±1.01 | 1.74±0.94 | 0.88±0.59 |
| Trincomalee | 2.37±1.55 | 2.11±1.46 | 1.32±1.15 | 2.63±1.63 | 7.90±2.83 | 2.30±1.50 | 1.52±1.21 | 4.46±2.06 | 3.40±1.78 | 4.75±2.08 | 1.64±1.22 | 0.23±0.45 | 0.23±0.44 | 1.80±1.25 | 2.03±1.33 | 2.58±1.46 |
| Kurunegala | 0.00±0.00 | 1.11±0.51 | 1.79±0.65 | 2.66±0.79 | 3.83±0.95 | 9.17±1.46 | 8.97±1.44 | 6.61±1.23 | 10.63±1.55 | 31.15±2.64 | 49.51±3.33 | 28.74±2.53 | 23.52±2.28 | 29.85±2.57 | 35.67±2.82 | 16.21±1.65 |
| Puttalam | 0.26±0.36 | 0.13±0.26 | 0.26±0.36 | 0.39±0.45 | 1.71±0.93 | 1.16±0.76 | 0.38±0.43 | 0.50±0.49 | 0.49±0.48 | 0.73±0.58 | 1.32±0.78 | 1.19±0.74 | 1.30±0.77 | 1.06±0.69 | 3.20±1.21 | 0.94±0.62 |
| Anuradhapura | 11.97±2.31 | 17.78±2.82 | 30.33±3.68 | 28.24±3.55 | 51.01±4.77 | 47.07±4.52 | 38.28±4.05 | 30.36±3.58 | 30.50±3.57 | 55.38±4.78 | 59.34±4.93 | 36.48±3.85 | 35.64±3.79 | 54.44±4.67 | 77.68±5.60 | 40.30±4.03 |
| Polonnarauwa | 2.22±1.45 | 5.42±2.26 | 20.93±4.45 | 20.44±4.40 | 43.83±6.44 | 36.54±5.81 | 30.57±5.28 | 31.78±5.34 | 38.05±5.82 | 60.32±7.29 | 73.18±7.99 | 85.33±8.60 | 115.18±9.94 | 120.27±10.14 | 99.10±9.25 | 52.21±6.30 |
| Badulla | 0.12±0.24 | 0.00±0.00 | 0.00±0.00 | 0.00±0.00 | 0.98±0.68 | 0.12±0.23 | 0.95±0.66 | 0.47±0.46 | 1.62±0.85 | 1.37±0.78 | 2.16±0.97 | 3.50±1.23 | 2.79±1.09 | 3.67±1.25 | 5.02±1.47 | 1.52±0.66 |
| Monaragala | 0.00±0.00 | 0.89±0.87 | 0.00±0.00 | 0.67±0.75 | 3.33±1.68 | 7.07±2.41 | 8.42±2.61 | 8.28±2.57 | 7.22±2.39 | 10.39±2.85 | 4.44±1.85 | 0.00±0.00 | 10.30±2.80 | 33.79±5.05 | 37.52±5.32 | 8.82±2.08 |
| Ratnapura | 0.37±0.36 | 0.09±0.18 | 0.83±0.54 | 1.10±0.62 | 1.65±0.76 | 3.05±1.03 | 1.69±0.76 | 0.09±0.17 | 1.91±0.80 | 19.17±2.52 | 16.31±2.31 | 13.23±2.08 | 10.34±1.83 | 19.78±2.52 | 18.86±2.47 | 7.23±1.26 |
| Kegalle | 0.00±0.00 | 0.12±0.23 | 0.00±0.00 | 0.24±0.33 | 0.24±0.33 | 0.23±0.32 | 0.00±0.00 | 0.34±0.39 | 1.37±0.77 | 1.92±0.91 | 7.89±1.85 | 5.84±1.59 | 3.56±1.23 | 3.79±1.27 | 5.72±1.57 | 2.08±0.72 |
| **Annual average** | 2.18±0.71 | 2.04±0.62 | 3.93±0.76 | 4.41±1.25 | 9.42±2.19 | 8.52±1.96 | 7.43±1.62 | 7.45±1.52 | 8.68±1.71 | 17.08±2.47 | 20.10±2.60 | 17.20±2.34 | 15.28±1.96 | 18.56±2.36 | 19.97±2.57 | 10.82±1.77 |

**Supplementary Table 2.** Annual incidence rates (per 100,000 population per year) of leishmaniasis in different districts of Sri Lanka, 2009–2023
