# Supplementary figures and images for: Trend analysis and spatiotemporal distribution of leishmaniasis disease incidence in Sri Lanka: A detailed review from 2009 to 2023
Source: PLoS Negl Trop Dis. 2025 Jul 2;19(7):e0013158. doi: 10.1371/journal.pntd.0013158 (PMC12221050; doi:10.1371/journal.pntd.0013158)

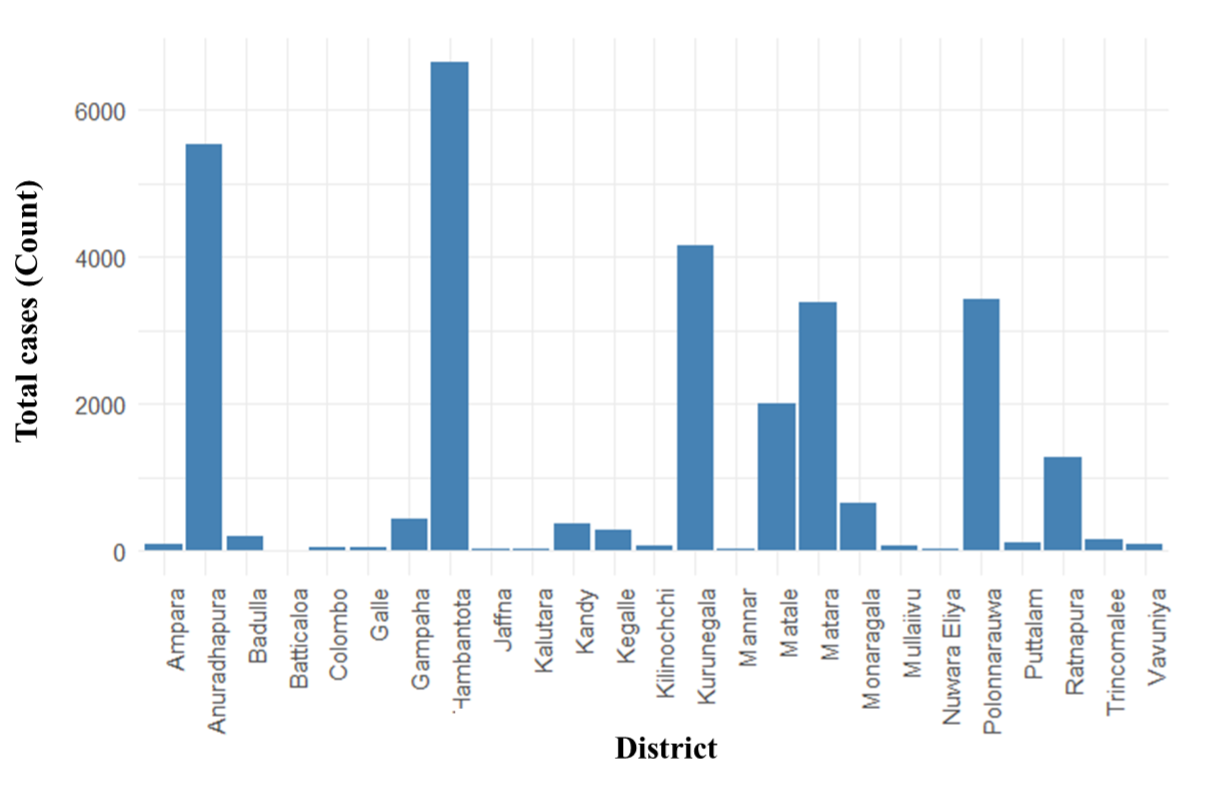

Supplement: S1 Fig — (TIFF) [file pntd.0013158.s003.tiff]
